# Chemico-Biological Profiling of *Blumea lacera* (Burm.f.) DC. (Family: Asteraceae) Provides New Insights as a Potential Source of Antioxidant, Cytotoxic, Antimicrobial, and Antidiarrheal Agents

**DOI:** 10.1155/2022/2293415

**Published:** 2022-08-12

**Authors:** Sania Ashrafi, Safaet Alam, Ariful Islam, Nazim Uddin Emon, Quazi Sufia Islam, Monira Ahsan

**Affiliations:** ^1^Department of Pharmaceutical Chemistry, University of Dhaka, Dhaka 1000, Bangladesh; ^2^Drugs and Toxins Research Division, BCSIR Laboratories Rajshahi, Bangladesh Council of Scientific and Industrial Research, Rajshahi 6206, Bangladesh; ^3^Department of Pharmacy, Faculty of Science and Engineering, International Islamic University Chittagong, Chittagong 4318, Bangladesh

## Abstract

*Blumea lacera* (Burm.f.) DC., popular for its traditional use in different diseases, was employed in phytochemical and biological investigations. The chemical studies led to the isolation of acyclic diterpene-phytol (1) along with two fatty acids-linolenic acid (2) and oleic acid (3). All the structures were determined by ^1^H NMR spectroscopic analysis and first time reported from this plant. Different fractions of crude methanol extract were subjected to antioxidant, cytotoxicity, antimicrobial, and antidiarrheal assays. The molecular docking studies have been implemented using PyRx, UCSF Chimera, Discovery Studio, and online tools. In addition, The ADME/T analysis and PASS prediction were implemented by using PASS online tools. In the molecular docking study of antioxidant, cytotoxicity, antimicrobial, and antidiarrheal activity, the compounds showed strong binding affinity ranging from −4.5 to −6.2 kcal/mol. Again, all three isolated compounds met the preconditions of Lipinski's five rules for drug discovery. In DPPH free radical scavenging assay, the pet-ether and chloroform soluble fraction showed noteworthy antioxidant activity sowing promising IC_50_ values (10.76 *μ*g/ml and 11.77 *μ*g/ml, respectively), compared to the standard (6.05 *μ*g/ml) with a total phenolic content range of 7.33–40.33 mg of GAE/gm. The pet-ether soluble fraction revealed substantial cytotoxicity showing an LC_50_ value of 1.03 *μ*g/ml, compared to the standard (0.93 *μ*g/ml). Besides, ethyl acetate soluble fraction showed moderate activity against both Gram-positive and Gram-negative bacteria, while both ethyl acetate and pet-ether soluble fraction showed excellent dose-dependent antidiarrheal activity.

## 1. Introduction

Plants generate a great variety of chemical constituents, called secondary metabolites that evidently have no obvious part in the growth and reproduction of plants. These chemical substances provide pharmacological action on the human system. The most effective of these biologically active compounds are flavonoids, alkaloids, phenolic compounds, and tannins [1]. So, isolation and purification of secondary metabolites from plants are now practiced extensively.

*Blumea lacera* (Burm.f.) DC., (Family: Asteraceae) is often locally called janglimulli, siyalmutra, susksampatra, and kakaronda. The plant has a camphor-like smell, a high stem, and is a corymbose pattern branched herb. It is commonly found growing in wastelands and roadside areas. It is commonly known that almost all parts of the plant (stem and leaves) possess active principles [2]. The chief secondary metabolites classes screened from this plant were alkaloids, amino acids, carbohydrates, tannins, phenolic compounds, reducing sugar, flavonoids, saponins, coumarin, and terpenoids [3]. Small amounts of campesterol, acetylenic compounds, a thiophene derivative, a diester, prenylated phenol glycosides, triterpenoid, flavonoids, and monoterpene glycosides have been reported to be present in the phytochemical investigation [4]. The Indian system of conventional medicine-Ayurveda describes its use as bitter, acrid, astringent, thermogenic, errhine, styptic, anti-inflammatory, digestive, ophthalmic, liver tonic, anthelmintic, febrifuge, expectorant, diuretic, antipyretic, stimulant, and deobstruent [5]. The methanolic leaf extract of *B. lacera* has been reported to reveal to have antifungal, antibacterial, cytotoxic, antipyretic, antiviral, antileukemic, and antidiarrheal activities [6].

Oxidative stress indicates the imbalance between the formation of reactive oxygen species (ROS) and the defense of the antioxidant system in favor of the former, where ROS causes oxidation of blood vessel walls, carbohydrates, DNA, and lipids [7]. Cancer is one of the primary reasons of mortality worldwide after cardiovascular diseases in severity. Each year, an average of 182 per 100 000 persons struggle with cancer in the world, and 102 people face death as a result of cancer. As per the report of the World Health Organization (WHO), the number of people burdened with cancer is 14 million and that of fatal cases is 8 million worldwide [8]. Reports say, in Bangladesh, 13% of death occurs due to cancer [9]. Antibiotic resistance, a burning issue nowadays in public health, develops due to the random usage of commercially available antibiotic drugs. Methicillin-resistant *Staphylococcus aureus* (MRSA) causes nearly 50,000 deaths each year in the United States and Europe alone, with so many deaths from it in further areas [10]. According to the Global Antimicrobial Resistance and Use Surveillance System (GLASS) report published by WHO in 2020, the frequency of antibiotic resistance occurs in 2,164,568 cases whereas 66 countries, territories, and areas were reported to have infectious diseases in 2018 [11]. Consequently, we need antibiotics with higher doses with compromised side effects. Herbal medicine can be a considerable option herein [12]. Diarrhea, a popular disease refers to an increased occurrence of fluid defecation accompanied by abdominal pain [13]. As stated by the UNICEF and WHO reports, the number of diarrheal cases that occur globally each year is 2.5 billion, and 1.9 million children below 5 years perish from diarrhea each year whereas most of the scenarios are from developing countries. 78% of all child deaths caused by diarrhea happen in Southeast Asian and African areas [14]. Numerous treatment approaches are available for the abovementioned conditions. Nonetheless, adverse effects, contraindications, non-selectivity of chemotherapeutic drugs, antibiotic resistance, toxic reactions, and high price limit the use of conventional treatment procedures. Hence, accessibility to natural products with greater efficiency and lesser adverse effects is anticipated.

Furthermore, plant-based bioactive phytochemicals can be selected from the online library and screened with vast numbers of target proteins using *in silico* techniques which can be beneficial in terms of both saving time and cost along with validation of acquired results from wet-lab experiments [15]. Consequently, most likely drug target and their responsible pathways to exert pharmacological actions can be forecasted with greater accuracy. The computational approach, on the other hand, is also playing a major part, as extensive data can be validated and generated by experimental and molecular biologists even without using wet labs. Thus, to ascertain and explore the drug design of a novel moiety, molecular docking and computer-aided drug discovery (CADD) approaches have been considered very popular options nowadays [16]. An effective molecular docking technique should be capable of identifying the local ligand pose along with the binding site of the 3D protein structure, as well as physical and chemical relationships [17]. Alongside molecular docking, toxicological evaluation of these novel moieties/phytochemicals based on Lipinski's rules can be a very handy tool to establish their drug-like characteristics [15].

In this study, we conducted our research on *B. lacera* and reported three compounds from the methanol extract of the whole plant. Besides, antioxidant, cytotoxicity, antimicrobial, and antidiarrheal activities of plant extracts and isolated phytochemicals were also evaluated and reported following *in vivo*, *in vitro,* and *in silico* approaches.

## 2. Materials and Methods

### 2.1. Sample Collection and Preparation

The whole plant including the root of *Blumea lacera* was collected from Gazipur, Bangladesh in May 2019. The identification of the plant was done by a professional from Bangladesh National Herbarium (BNH) and a specimen voucher for this collection was submitted for future reference with accession number 55315. The plant parts were cleaned properly. After cutting the plant parts into small pieces they were exposed to shade drying for 7 days and the dried material was crumbled into coarse powder by careful use of the high-quality grinding machine. The final product sample was 750 g.

### 2.2. Instrumentations, Drugs, and Chemicals

Bruker (400 MHz) instrument was used to record NMR spectra in deuterated chloroform (CDCl_3_). Solvent evaporation was done by Buchi Rotavapor (Germany). Kieselgel 60H and Sephadex LH 20 (Sigma-Aldrich, USA) were used to perform Vacuum Liquid Chromatography (VLC) and Gel Permeation Chromatography (GPC), respectively. Analysis of the compounds was performed on precoated thin layer chromatography plates (Silica gel 60 F 254, Merck, Germany). UV light and vanillin/H_2_SO_4_ reagents were used for the visualization of the spots on TLC plates. All the other reagents and solvents consumed in the research were of analytical grade and obtained from a reliable source (Active Fine Chemicals Ltd, Bangladesh; Merck, Germany; DaeJung, Korea). Loperamide and vincristine sulfate used in the study was obtained from Opsonin Pharma Ltd., Bangladesh.

### 2.3. Test Microorganism

Gram-positive bacteria (*Sarcina lutea* and *Staphylococcus aureus*) and Gram-negative bacteria (*Salmonella typhi*, *Escherichia coli*, *Salmonella paratyphi*, *Shigella flexneri*, and *Klebsiella* spp.) were utilized for the antimicrobial assay, which was provided from University of Dhaka, Bangladesh.

### 2.4. Experimental Design

#### 2.4.1. Extraction of Plant Material

750 g of the air-dried and powdered material of *Blumea lacera* was taken in a clean, amber-colored bottle and drenched in distilled methanol (MeOH), for 2 weeks with occasional stirring and shaking. After two weeks of cold extraction, the mixture was filtered by using a cotton plug in the large funnel and using a Buchii Rotavapour (Germany), and the volume of the filtrate was reduced. This process was done multiple times over 6 days and dried extracts were collected in the same beaker. Finally, 25 g (3.33%) of dried methanol extract was obtained.

#### 2.4.2. Isolation of Compounds

Vacuum Liquid Chromatography (VLC) was performed on a part of the crude extract (20 g) over silica gel [18] using hexane, ethyl acetate (EtOAc), and MeOH with increasing polarity. Altogether 36 VLC fractions were collected. VLC fraction 11–14 (80% hexane in chloroform) was fractionated on a Sephadex LH-20 column into 20 fractions each using CHCl_3_ as the eluting solvent. Sephadex fractions 18–29 of VLC fraction 11–12 yielded compound 1 and Sephadex fractions 1–3 of VLC fraction 13–14 yielded compounds 2 and 3.

#### 2.4.3. Test Animal Model

Male and female Swiss Albino mice (average weight 25–30 gm, aged 4–5 weeks) were obtained from the International Centre for Diarrheal Diseases and Research, Bangladesh (ICDDR, B) animal branch and were reserved in polypropylene-made crates in a controlled room (12 hr light-dark cycle; relative humidity 60–70%; temperature 24 ± 2°C). They were given rodent food formulated by iccdr, b, and water *ad libitum*. They were kept in the experimental conditions for a week, to cope with the environment, before the test. The guidelines of the Federation of European Laboratory Animal Science Associations (FELASA) were followed throughout the experiment. Guidelines for the care and use of laboratory animals approved by the Institutional ethical committee were followed while conducting all the experiments [19].

#### 2.4.4. Acute Toxicity Test

A single oral dose of either 500 mg/kg b.w., 1000 mg/kg b.w., 1500 mg/kg b.w., or 2000 mg/kg b.w. of *B. lacera* extract was given to a total of 25 Swiss albino mice aged 4 to 5 weeks. Feeding was stopped for 3–4 h. After the doses were given, all animals were preserved under close monitoring for 30 min intermittently for the first 24 h for consecutive 3 days to observe any delayed toxicity as well as fluctuations in respiratory and circulatory rate, skin and fur, eyes, and mucous membranes, or autonomic and CNS function. The reported protocol of Test No. 423 (OECD, 2001; Acute oral toxicity-acute toxic class method) was followed for this experiment [20].

#### 2.4.5. Preparation of Different Partitions for Biological Tests

The protocol outlined by Kupchan and improved by Van Wagenen et al. [21] was used for solvent-solvent partitioning. The crude MeOH extract was fractionated with pet-ether, then with chloroform, and lastly with ethyl acetate. These fractionate were then evaporated in a rotary evaporator separately to bring out petroleum ether soluble fraction (BLP, 1.9 g), chloroform soluble fraction (BLC, 1.4 g), ethyl acetate soluble fraction (BLE, 1.1 g), and aqueous soluble fractions (BLA, 0.9 g).

#### 2.4.6. Structural Identification of the Compounds

Bruker 400 NMR spectrometer was used to measure ^1^H NMR spectra of compounds 1–3, in deuterated chloroform (CDCl_3_), at 400 MHz and the *δ* values are described relative to the residual non-deuterated solvent signal. Coupling constants are given in Hertz (Hz). The chemical shifts are expressed in *δ*ppm.

### 2.5. Antioxidant Assay

#### 2.5.1. DPPH Free Radical Scavenging Assay

2.0 ml of methanol solution of the extract at serially diluted different concentrations (200 *μ*g/mL to 0.78125 *μ*g/mL) were mixed with 3.0 ml of a DPPH methanol solution (20 *μ*g/ml) to assess the free radical scavenging activities of the plant extracts on 1, 1-diphenyl-2-picrylhydrazyl (DPPH). From the decolorizing of purple-colored DPPH methanol solution by the plant extract compared to that of Butylated Hydroxy Anisole (BHA) by UV spectrophotometer, the antioxidant activities were evaluated [22].

(1) $$\begin{array}{c} \%\text{Inhibition of free radical }DPPH=\left(1-\frac{\text{Absorbance of sample}}{\text{Absorbance of the control reaction}}\right)\times \;100. \end{array}$$

#### 2.5.2. Total Phenolic Content

Folin–Ciocalteu Reagent (FCR) was used as an oxidizing agent and gallic acid as the reference standard to quantify the total phenolic content by following the procedure of Harbertson and Viticulture [23]. A total of 2.5 ml of FCR and 2 ml of Na_2_CO_3_ were mixed with 0.5 ml of extract solution (2 mg/ml), incubated for 20 min at ambient temperature, and then the absorbance values were recorded by a UV spectrophotometer at 760 nm and by analyzing the standard curve made of gallic acid at different concentrations, the total phenol content values were calculated. The phenolic contents were reported as milligrams of gallic acid equivalent (GAE)/gram of extract.

### 2.6. Cytotoxicity Assay

#### 2.6.1. Brine Shrimp Lethality Bioassay

To assess the cytotoxic potentials of different extracts of the plant, a brine shrimp lethality bioassay was carried out. For the simulation of seawater, 38 g of NaCl salt was dissolved in 1000 mL of distilled water with the addition of NaOH to maintain pH (8.0). To get nauplii, brine shrimp eggs were hatched in simulated seawater. Test samples were prepared by mixing 0.1 *μ*g/ml of dimethylsulphoxide (DMSO) with serially diluted different concentrations (400 *μ*g/mL to 0.78125 *μ*g/mL) of plant extract. Vincristine sulfate at different concentrations (400 *μ*g/mL to 0.78125 *μ*g/mL) was used as the reference standard and DMSO was used as a negative control. The nauplii were counted by visual check and mixed with 5 ml of simulated seawater in vials at ambient temperature (25 ± 1°C). Then by using a micropipette, samples of different concentrations were added to the premarked vials. After 24 h, survivors were counted [24].

(2) $$\begin{array}{c} \text{Mortality}\left(\%\right)=\frac{\text{Number of nauplii death}}{\text{Number of nauplii taken}}\times 100. \end{array}$$

### 2.7. Antimicrobial Assay

#### 2.7.1. Disc Diffusion Test

To assess the antimicrobial potential of different fractionates of the crude extract, the disc diffusion method was performed [25]. In this conventional method, desiccated and sterilized filter paper discs (6 mm) with the test samples were uniformly spread on the nutrient agar medium which was preinoculated with test bacteria. Four antibiotic discs (Vancomycin, Azithromycin, Tetracycline, and Levofloxacin) available in the market were considered as positive standard blank discs as negative standard. The plates were preserved at a low temperature (4°C) for about 24 hours upside down to facilitate maximum diffusion of test samples into the medium. Then the plates were upturned and reserved in an incubator at 37°C for 24 h. The samples having antimicrobial potential diffused profoundly through the medium and inhibited the growth of microorganisms, which was visible as a clean, separate area marked as a zone of inhibition. The diameters of the zones of inhibition were measured in millimeters to estimate the antimicrobial activities of those test samples.

### 2.8. Antidiarrheal Assay

#### 2.8.1. Castor Oil-Induced Diarrhea Test

*In vivo*antidiarrheal test was carried out by following the castor oil-induced method mentioned by [16]. Experimental albino mice were selected by giving them 0.5 ml of castor oil and just those who developed diarrhea were chosen for the study. At Random, four groups were created containing three mice in each group to be allotted as test animals and they were kept in fasting conditions for 24 hours before the test with free access to water. Group 1 animals were designated as a control group and were given 1% Tween 80 in distilled water. Group 2 was named as positive or standard and loperamide (2 mg/kg b. w. i.p) which was used as a reference standard was given to the group. group 3 and group 4 were given the different fractions of the crude extract at doses of 200 and 400 (mg/kg of b.w.), respectively. 1 h after administering the test samples, all the mice were given 0.5 mL of castor oil and they were positioned distinctly on the box floor with transparent paper. While on observation, the onset of diarrhea, quantity, and weight of wet stools, and total quantity and the total weight of fecal yields were documented.

(3) $$\begin{array}{c} \%\text{Inhibition of defecation}=\frac{\text{Mean number of wet feces by control }-\text{Mean number of wet feces by test sample or standard}}{\text{Mean number of wet feces by control}1}\times 100. \end{array}$$

### 2.9. *In Silico* Studies

#### 2.9.1. Molecular Docking: Ligand Preparation

From the PubChem database, three aforementioned chemicals' structures of *B*. *lacera* plant extracts, Linolenic Acid (PubChem CID: 5280934), Oleic Acid (PubChem CID: 445639), and Phytol (PubChem CID: 5280435), has been obtained. The ligands have been downloaded in 2DSDF format and then modified and processed to PDBQT format using PyRx tools to attain a better optimal hit against those target targets. For virtual screening, PyRx from MGL Tools (https://ccsb.scripps.edu/mgltools/) has been used with default parameters [26].

#### 2.9.2. Molecular Docking: Protein Preparation

3D crystal structures of target proteins including M3 muscarinic cholinergic receptor (PDB ID: 5ZHP) [27], Glutaminase domain of glucosamine 6-phosphate synthase (PDB ID: 1XFF) [28], urate oxidase (PDB ID: 1R4U) [29], and crystal structure of human cytochrome P450 2C9 (PDB ID: 10G5) [30] was obtained in PDB format from the RCBS Protein Data Bank (https://www.rcsb.org/structure). During the evaluation, Discovery Studio 2020 was used to eliminate all water and heteroatoms from the target proteins. The Gasteiger charge was assigned to proteins after nonpolar hydrogens were combined. Furthermore, in UCSF Chimera, all proteins were reduced to their lowest energy level by preserving standard residues in AMBER ff14sB and other residues in Gasteiger mode and then analyzed for further evaluation [31].

#### 2.9.3. Molecular Docking Analysis

PyRx AutoDock Vina was applied to link the proteins and ligands in the desired protein-ligand complexes. A semi-flexible docking approach was considered in the docking analysis. PDB files of phytocompounds and target proteins were converted to PDBQT format employing the PyRx AutoDock program [32]. The stiffness of proteins and the flexibility of ligands were both retained in this investigation. Ligand molecules had a total of ten degrees of freedom. AutoDock outlines the processes for converting biomolecules to PDBQT format, sorting boxes, creating grid boxes, and so on. The grid box with an active site was created in the center of the box. Lastly, BIOVIA Discovery Studio visualizer 2020 has been speeded up to evaluate docking spots for the most effective connecting strategies [26].

### 2.10. ADME/T Analysis of Ligands

#### 2.10.1. Prediction of Absorption, Distribution, Metabolism, and Excretion by SwissADME

SwissADME online tool has been utilized in this study to determine the molecular weight of the isolated phytochemicals along with their lipophilicity (LogP) parameter, hydrogen bond acceptors' numbers, and hydrogen bond donors' numbers based on the rules of Lipinski to explore the pharmacokinetic profile or drug-likeness characteristics of the isolated phytochemicals [16].

#### 2.10.2. Prediction of Toxicological Properties by Admet SAR

Since toxic effects of prospective moieties are a major concern during the drug development procedure, the admetSAR online tool (https://lmmd.ecust.edu.cn/admetsar1/predict/) has been adopted in this study to determine the toxicological profile of the isolated phytochemicals. AMES toxicity, hydrogen bond accepting and donating profile, carcinogenic attributes, lipophilicity, blood-brain barrier permeability, and acute rat toxicity profile were all estimated in this study.

## 3. Results

### 3.1. Isolated Phytochemicals from *B*. *lacera*

**Figure 1:**
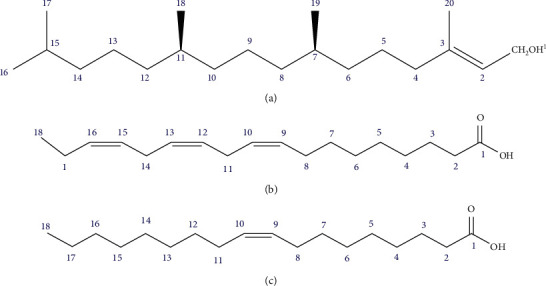
The structures of isolated phytochemicals from *Blumea lacera* using NMR techniques: (a) phytol, (b) linolenic acid, (c) oleic acid.

Three compounds (Figure 1) were isolated from the crude methanol extract of the whole plant of *B.lacera* by following repeated chromatographic separations. The structures of the isolated compounds were elucidated as phytol (1) [33], linolenic acid (2) [34], and oleic acid (3) [35] by analyzing the NMR spectral data and comparing those data with published values.

Phytol ((2E, 7R, 11R)-3,7,11,15-tetramethyl-2-hexadecen-1-ol) (1): Colorless mass and soluble in ethyl acetate and chloroform; ^1^H NMR (400 MHz, CDCl3): *δ*4.17 (2H d, *J* = 6.8 Hz, H-1), 5.43 (1H t, *J* = 6.6 Hz, H-2), 2.01 (2H t, *J* = 7.2 Hz, H-4), 0.88 (3H d, *J* = 6.8 Hz, H-16), 0.88 (3H d, *J* = 6.8 Hz, H-17), 0.86 (3H d, *J* = 6.0 Hz, H-18), 0.85 (3H d, *J* = 7.0 Hz, H-19), and 1.69 (3H s, H-20).

Linolenic acid (cis, cis-9,12-Octadecadienoic acid) (2): Light yellowish liquid and soluble in ethyl acetate and chloroform; ^1^H NMR (400 MHz, CDCl3): *δ*2.37 (2H t, *J* = 7.4 Hz, H-2), 1.64 (2H m, H-3), 1.28–1.34 (8H m, H-4 to H-7), 2.07 (4H m, H-8, 17), 5.38 (6H m, H-9, 10, 12, 13, 15, 16), 2.80 (4H m, H-11, H-14), and 0.90 (3H t, *J* = 7.6 Hz, H-18).

Oleic acid ((9Z)-Octadec-9-enoic acid) (3): Light yellowish liquid and soluble in ethyl acetate and chloroform; ^1^H NMR (400 MHz, CDCl3): *δ*2.37 (2H t, *J* = 7.4 Hz, H-2), 1.64 (2H m, H-3), 1.28–1.34 (22H m, H-4 to H-7, H-12 to H-17), 2.07 (4H m, H-8, 11), 5.38 (2H m, H-9, 10), and 0.90 (3H t, *J* = 7.6 Hz, H-18).

Compound 1 was collected as a colorless mass. ^1^H NMR spectrum (400 MHz, CDCl_3_) displayed an oxygenated methylene doublet at *δ* 4.17 (2H d, *J* = 6.8Hz), an olefinic proton at *δ* 5.43 (1H t, *J* = 6.6Hz), and a deshielded methyl at *δ* 1.69 (3H s), which could be assigned to H-1, H-2 and H-20 of an acyclic diterpene, phytol. In addition, the spectrum showed four methyl doublets at *δ* 0.88 (*J* = 6.8 Hz), 0.88 (*J* = 6.8 Hz), 0.86 (*J* = 6.0 Hz), and 0.85 (*J* = 7.0 Hz) assignable to H-16, H-17, H-18, and H-19. A broad triplet at *δ* 2.01 (2H t, *J* = 7.2 Hz) was assigned as H-4. The ^1^H NMR data were found to be in close agreement with those published for the compound [33].

The ^1^H NMR spectrum (400 MHz, CDCl_3_) of compounds 2 and 3 came out as a mixture of two fatty acids-linolenic acids (2) and oleic acid (3). The six olefinic proton multiplets at *δ* 5.38 and a methyl triplet at *δ* 0.90 could be assigned to three conjugated doublets at C-9, C-12, C-15, and the terminal methyl group at C-18 respectively. The bis-allylic protons at positions C-11 and C-14 (=CH-CH_2_-CH=) appeared at *δ* 2.80 (4H m) and the protons resonated at *δ* 2.07 (4H m) are the allylic protons (CH_2_-CH = CH) of C-8 and C-17. The protons directly adjacent to the carbonyl group resonate at *δ* 2.37 (2H t, *J* = 7.4 Hz, H-2), and the CH_2_-CH_2_-COOH protons resonated at *δ* 1.64, (2H m, H-3). The methylene protons of the fatty chain appeared at *δ* 1.28–1.34 (8H m). The NMR spectral data were in agreement with literature values for linolenic acid [34].

The remaining signals of the spectrum, like two olefinic protons at *δ* 5.38 (H-9 and H-10), a terminal methyl at *δ* 0.90 (H-18), four allylic protons at *δ* 2.07 (H-8 and H-11), a methylene triplet attached to the carbonyl group at *δ* 2.37 (H-2), a slightly deshielded methylene resonating at *δ* 1.64, (H-3) and ten methylene protons of fatty chain at *δ* 1.28–1.34 (H-4 to H-7 and H-12 to H-17) suggested the structure of compound 3 as oleic acid. The structure was further confirmed by comparing its spectral data with those published [35].

### 3.2. Total Phenolic Content

The quantity of total phenolic content of different fractions was found to be in the range of 7.33–40.33 mg of GAE/gm of extractives (Table 1). The chloroform soluble fraction of the plant (BLC) showed the highest amount of phenolic content though Ethyl acetate soluble (BLE) fraction showed a considerable amount.

### 3.3. Effect of *B*. *lacera* Extracts on DPPH Free Radical Scavenging Activity

**Figure 2:**
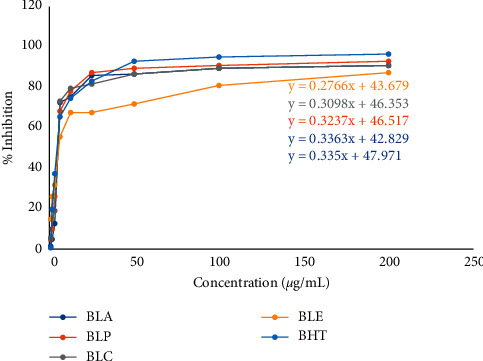
The IC_50_ value of butylated hydroxytoluene (BHT) and different extracts of *B*. *lacera*.

**Figure 3:**
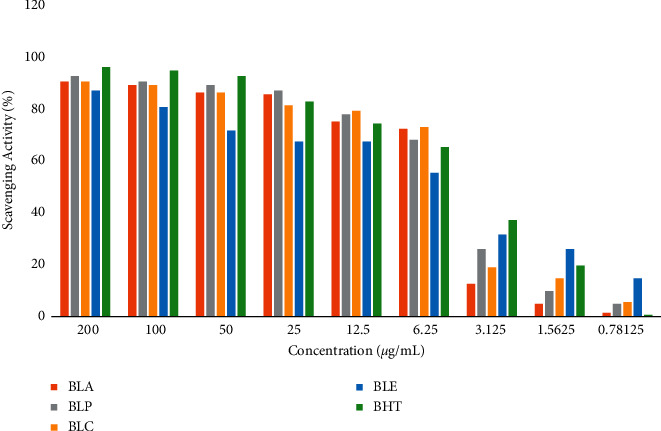
The percentage of radical scavenging activities of different extracts of *B*. *lacera*.

In the DPPH free radical scavenging study, different extracts of *B*. *lacera* exhibited a dose-dependent free radical scavenging activity in comparison with the standard. BLP showed substantial scavenging activity (92.96%) compared to the standard BHT (96.48%) at 200 *μ*g/mL. The IC_50_ values of BHT and the fractions were calculated by the linear regression equation, summarized in Figures 2 and 3.

### 3.4. Effect of *B*. *lacera* Extracts on Brine Shrimp Lethality Bioassay

**Figure 4:**
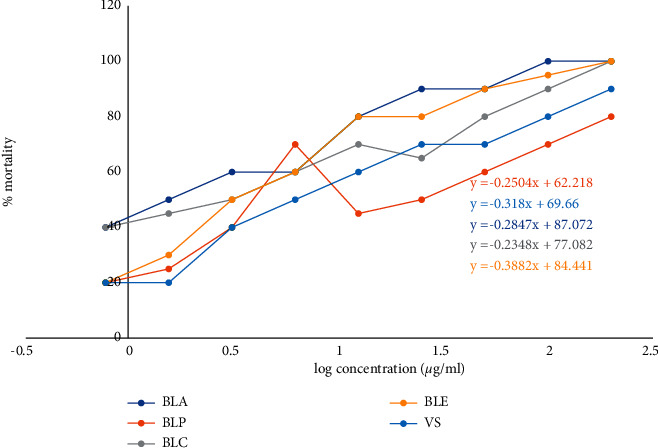
% Mortality and predicted regression line of vincristine sulfate and different extracts of *B*. *lacera*.

In the brine shrimp lethality test, different extracts of *B*. *lacera* displayed dose-dependent mortality compared to standard. BLP showed potent cytotoxicity with an IC_50_ value of 1.03 compared to that of standard (Figure 4) (Table 2).

### 3.5. Effect of *B. lacera* Extracts on Disc Diffusion Assay

All the partitions were assayed for the antimicrobial activities against two Gram-positive and four Gram-negative bacteria taking antibiotics vancomycin, azithromycin, tetracycline, and levofloxacin as standards (Table 3). The zone of inhibition of different extractives ranged from 5 mm to 7 mm. In this study, BLE showed inhibitory activity against *Staphylococcus aureus*, *Sarcina lutea*, *Salmonella typhi*, and *Escherichia coli*, while BLC showed antimicrobial activity against *Klebsiella* spp. and *Escherichia coli* making them a potential source for antimicrobial agents.

### 3.6. Effect of *B. lacera* Extracts on Castor Oil-Induced Diarrhea

After administering BLE, BLP, BLC, and BLA (at 200 and 400 mg/kg) significant (*p* < 0.05, *p* < 0.01, *p* < 0.001) a dose-dependent delay in the onset of diarrhea was observed while compared with the control. Likewise, a reduction in the number of wet feces, total number of feces, weight of wet feces, and total weight of all feces were also detected. The summary of castor oil-induced diarrhea has been shown in Table 4. In terms of wet feces number, the data revealed that the percentages of diarrhea inhibitions by 200 mg/kg dose were 57.96%, 54.86%, 41.75%, and 28.63% demonstrated by respectively BLE, BLP, BLC, and BLA at doses of 200 mg/kg while at 400 mg/kg the values were 64.36%, 61.17%, 44.95% and 32.03% demonstrated by, respectively, BLE, BLP, BLC, and BLA. Besides, loperamide showed inhibitory actions by 74.01%.

### 3.7. Molecular Docking Studies for Pharmacological Actions

**Figure 5:**
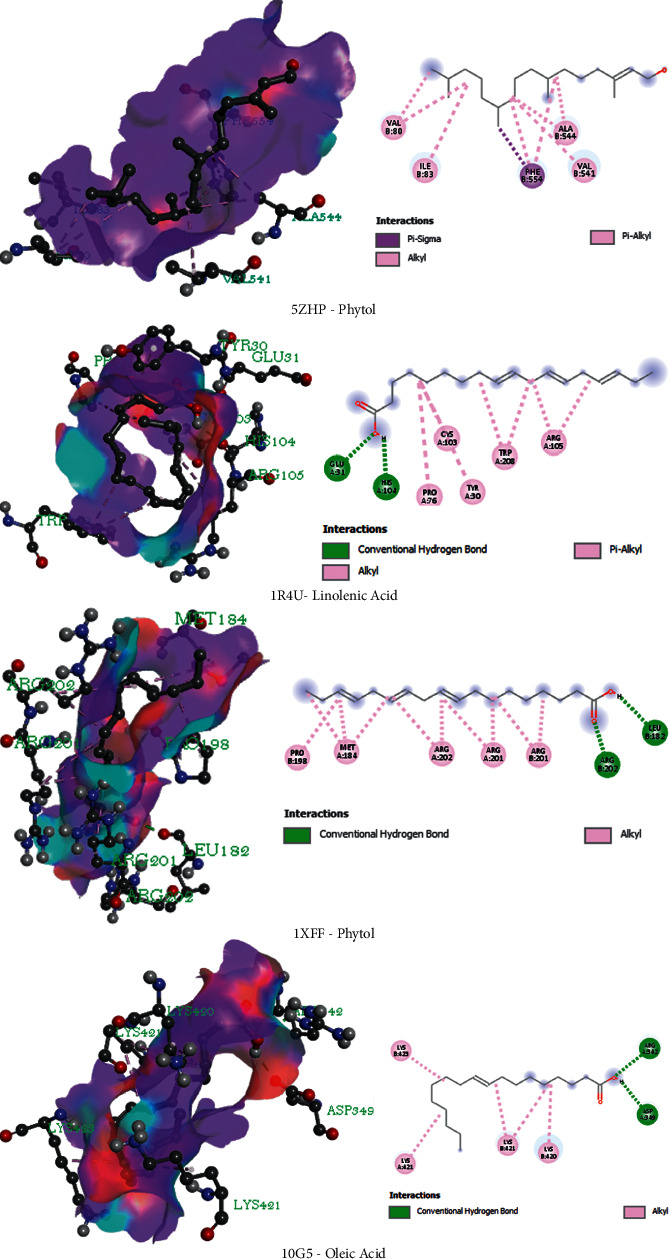
3D and 2D representations of the best key interaction between isolated phytochemicals and selected enzyme (Phytol with M3 muscarinic acetylcholine receptor, linolenic acid with urate oxidase, phytol with glutaminase domain of glucosamine 6-phosphate synthase and oleic acid with the crystal structure of human cytochrome P450 2C9).

**Figure 6:**
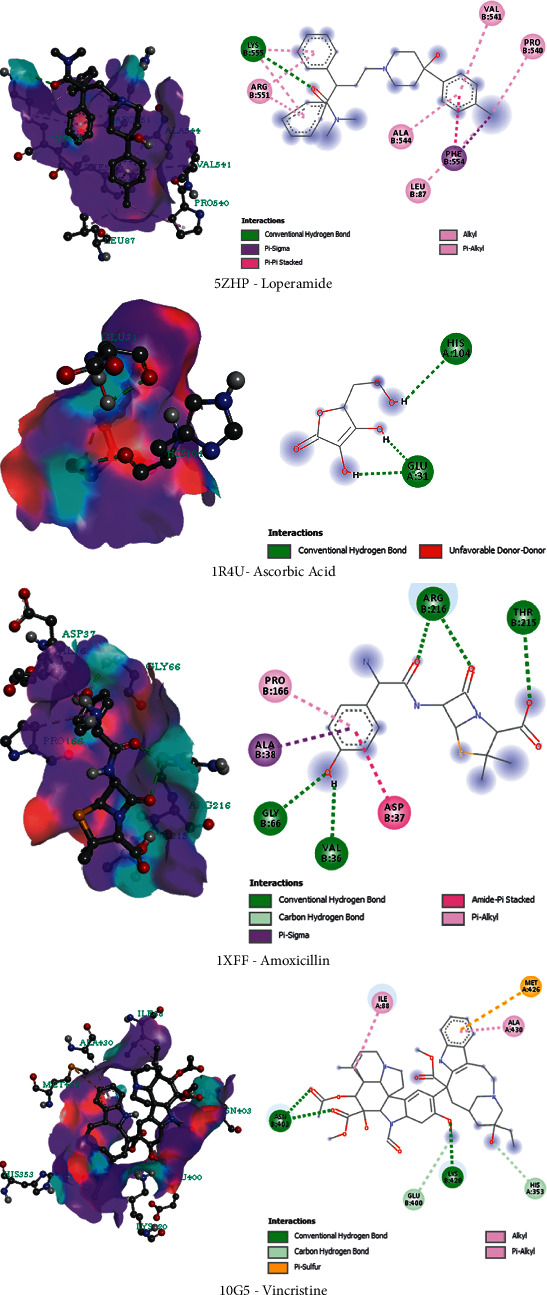
3D and 2D representations of the best key interaction between standard drugs and selected enzymes (loperamide with M3 muscarinic acetylcholine receptor, ascorbic acid with urate oxidase, amoxicillin with glutaminase domain of glucosamine 6-phosphate synthase and vincristine sulfate with the crystal structure of human cytochrome P450 2C9).

The antidiarrheal docking studies have been performed among the isolated compounds and M3 muscarinic acetylcholine receptor (5ZHP). The binding scores are −5.3 kcal/mol, −4.9 kcal/mol, and −5.6 kcal/mol for the linolenic acid, oleic acid, and phytol, respectively. Phytol showed maximum binding affinity via the interaction with a series of amino acids namely: val80, ile83, phe554, ala541, and val544. The docking of isolated phytochemicals with urate oxidase was used to conduct an antioxidant docking evaluation. (PDB ID: 1R4U). The docking score findings are reported in Table 5. Moreover, the docking interaction is also depicted in Figure 5. Based on the findings, the conducted study concluded that linolenic acid had the highest binding affinity to the 1R4U receptor and showed a docking score of −5.4 kcal/mol by interacting with a series of bonds namely: glu31, His104, pro76, cys103, tyr30, trp208, arg105. Besides, molecular docking studies of the isolated compounds on the antibacterial and cytotoxic activity revealed a strong binding affinity to the 1XFF and 10G5 receptors subsequently. The ranking of binding affinity towards 1XFF and 10G5 receptors are Phytol > Linolenic Acid > Oleic Acid and Oleic Acid > Linolenic Acid > Phytol, respectively. In addition, the binding affinity of the standard drugs has been also summarized in Figure 6 and Table 5.

### 3.8. Pharmacokinetic (ADME) and Toxicological Properties Prediction

Lipinski used SwissADME, an online tool, to determine the pharmacokinetic properties of the substances he chose. Lipinski has stated that a substance can be orally bioavailable if it meets the following criteria: molecular weight of less than 500 amu, hydrogen bond donor sites of less than 5, hydrogen bond acceptor sites of less than 10, and Lipophilicity value (LogP) of less than or equal to 5. The research concluded that all of the isolated phytochemicals followed Lipinski's rules, indicating that they have a high oral bioavailability (Table 6). The toxicological properties of the isolated phytochemicals were also projected by the admetSAR online server. The isolated phytochemicals were found to be non-Ames toxic, non-carcinogenic, and have low rat toxicity parameters.

## 4. Discussion

Plant-based drugs and chemicals are being used to treat different illnesses since the emergence of human civilization. Since ancient times, plants are being used extensively by all cultures for the improvement of health and as a treatment procedure for various diseases. As stated by World Health Organization (WHO), 80% of world residents are dependent on traditional medicine to meet their principal health care [36, 37]. Many plant-derived secondary metabolites have been found to act as thrombolytic, antioxidant, antidepressants, cytotoxicity, anxiolytics, neuroprotective, and hepatoprotective agents [38].

Antioxidants are substances that can cause reactive oxygen species (ROS) scavenging [39]. They play a vital role in the human body to reduce oxidative stress and the destructive effects of ROS [40]. Multiple reports say that plant phenolics are the most significant group of secondary metabolites that play a role as primary antioxidants or free radical scavengers and flavonoids and tannins are the phenolic compounds [41]. The existence of flavonoids and tannins in *B*. *lacera* is accountable for the free radical scavenging effects. In the quantitative test of BLC, the total phenol content was found 40.33 mg GAE/g. Besides, from the DPPH scavenging test, BLP showed outstanding antioxidant activity with an IC_50_ value of 10.76 *μ*g/mL while standard BHT revealed an IC_50_ value of 6.05 *μ*g/mL. Cancer is a crucial health issue in both developed and developing countries. The presence of flavonoids is also accountable for showing antitumorigenic activity in different stages of cancer growth and development of cancer following diverse mechanisms including induction of apoptosis in tumor cells [42]. The cytotoxic activities of BLP (LC_50_ 1.03) compared to vincristine sulfate (LC_50_ 0.93) may occur due to the presence of diterpene, alkaloid, and steroids [41]. Bacteria have a genetic tendency to develop resistance [43]. Hence, the necessity to develop newer antimicrobial drugs to fight microbes of numerous spectra, even if pharmaceutical industries have developed varieties of antibiotics [44]. BLE showed potent antimicrobial activities, probably for the presence of numerous phenolic compounds [3]. Being one of the principal reasons for inevitable death in developing countries, diarrhea primarily affects children as well as infants. The active component of castor oil, ricinoleic acid is well-known to accelerate peristalsis in the small intestine which causes inflammation of the mucosa of the intestine attributable to prostaglandins secretion while changing the water, electrolyte permeability of the intestinal wall; augmenting diarrhea [45]. The antidiarrheal effect of *B*. *lacera* could be due to the prevalence of numerous phytoconstituents like flavonoids and tannins. Some alkaloids, mono, di, tri terpenoids, and cardiac glycosides present in the plants may also be responsible for the antidiarrheal activities in the animal model [5, 46–49]. These findings validate the extensive traditional use of this plant. Furthermore, molecular docking studies have been employed thoroughly to estimate ligand-target associations and attain a better interpretation of the pharmacological attributes of isolated phytochemicals. It offers extensive insights into the prospective mechanisms of action and binding profile of several proteins and concerned phytochemicals within their binding pockets. Three isolated phytochemicals from *B*. *lacera* were chosen for docking experiments to gain a better understanding of their pharmacological actions (antioxidant, cytotoxicity, antimicrobial, and antidiarrheal). In humans, muscarinic M (3)-specific receptor antagonists are thought to slow gastrointestinal and colonic transit. The findings show that when muscarinic antagonists are taken at clinically approved levels, muscarinic M (3) receptors govern small intestine and colonic transit in humans [50]. The amide nitrogen of glutamine is used by glutamine amidotransferases (Glutaminase domain of glucosamine 6-phosphate synthase) in the production of amino acids, nucleotides, and amino sugars. They are made up of two domains: a synthetase (or synthase) domain that links the nitrogen acceptor and a glutaminase domain that catalyzes glutamine hydrolysis to glutamate and ammonia. Within each of the two groups of amidotransferases, the glutaminase domain is substantially preserved. The discovery of new therapeutic molecules that specifically inhibit the catalytic properties of microbial enzymes could have significant implications for the invention of novel therapeutics to halt the catalytic activity of these enzymes, given their central role in cellular metabolism [28]. Uric acid, a powerful plasma antioxidant that scavenges singlet oxygen, peroxy radicals, and hydroxyl radicals, has been thoroughly researched in a variety of physiological and pathological systems, including neurological disorders. Uric acid (urate oxidase) can also bind to iron ion complexes, suggesting that it has further antioxidant properties [51]. Uric acid showed a concentration-dependent effect on decreasing hydroxyl and superoxide radicals, meaning that increasing uric acid concentration enhanced radical scavenging [52]. The CYP2C9 (human cytochrome P450 2C9) metabolite 16-hydroxyestradiol is linked to a higher risk of breast cancer. Increased levels of 16-hydroxyestradiol can significantly increase the risk of breast cancer, as reported in epidemiological case-control research and prospective analysis. In the arachidonic acid metabolic pathway, CYP2C19 is a crucial enzyme in the synthesis of epoxy-eicosatrienoic acids (EETs). During both in vivo and in vitro studies, exogenous EET administration stimulates cancer cell proliferation. EETs activate the MAPK and PI3K/Akt signaling pathways, enhance EGFR phosphorylation, regulate the tumor microenvironment, and facilitate immunosuppression in a number of tumor cell lines following an autocrine and paracrine manner [53]. The docking scores for the concerned phytochemicals ranged from −4.5 to −7.2 kcal/mol, indicating that they functioned with enzymes via several amino acid residues, which is essential for pharmacological actions. The binding affinities of standard drugs were compared to the docking results of concerned phytochemicals. These findings suggest that the isolated phytochemicals can be the driving force of antioxidant, cytotoxic, antibacterial, antidiarrheal, and thrombolytic properties of *B*. *lacera*. All phytochemicals were investigated for drug-like attributes, pharmacokinetics, and physicochemical properties using the online prediction tool ADME. All isolated phytochemicals of *B*. *lacera* evidenced orally active drug-like qualities, according to the rules of Lipinski. High permeability, adequate absorption, and greater bioavailability can be claimed for phytochemicals with a lower molecular weight, lipophilicity, and hydrogen bonding profile [54].

## 5. Conclusion

In this experiment, BLP showed the maximum free radical scavenging and cytotoxic activities, while both BLE and BLP showed significant antidiarrheal activities. Again, BLE exhibited antimicrobial activity against *Staphylococcus aureus*, *Sarcina lutea*, *Salmonella typhi*, and *Escherichia coli,* and BLC against *Klebsiella* spp., and *Escherichia coli.* The methanol extract of the whole plants of *B*. *lacera* upon successive chromatographic separation and purification yielded a total of three compounds, which can be responsible for our observed pharmacological actions as computer-aided approaches also support the drug-like properties of those isolated phytochemicals. Extensive investigations on this plant are still recommended to isolate other bioactive phytochemicals and determine their exact mode of action to exert a pharmacological response along with their safety profile.

## Figures and Tables

**Table 1:** The quantitative analysis of total phenol content of *B*. *lacera* extracts.

Plant extract	Total phenolic content (mg of GAE/gm of extract)	Regression line
BLA	7.33	*y* = 0.0076x − 0.0064, *R*^2^ = 0.9993
BLP	19.65	
BLC	40.33	
BLE	35.24	

**Table 2:** LC_50_ values of vincristine sulfate different extracts of *B*. *lacera*.

Plant extract	VS	BLA	BLP	BLC	BLE
LC_50_ values	0.93	0.12	1.03	0.41	0.58

**Table 3:** The antimicrobial activity of *B*. *lacera* extracts and standard against Gram-positive and Gram-negative bacterial strains.

Test microorganisms	Diameter of zone of inhibition (mm)				Vancomycin	Azithromycin	Tetracycline	Levofloxacin
	BLA	BLP	BLC	BLE				
*Gram-positive bacteria*								
*Staphylococcus aureus*	—	—	—	7	10	16	16	17
*Sarcina lutea*	—	—	—	7	14	14	15	16

*Gram-negative bacteria*								
*Salmonella typhi*	—	—	—	5	12	6	10	4
*Klebsiella* spp.	—	—	5	—	15	7	8	13
*Shigella flexneri*	—	—	—	—	18	8	9	6
*Escherichia coli*	—	—	6	7	14	5	7	11

**Table 4:** The antidiarrheal effect of different extracts of *B*. *lacera* leaves on castor oil-induced test in mice.

Doses (ml/kg, b.w; p.o)	Onset of diarrhea	Average number of wet feces	Average number of total feces	Average weight of wet feces (g)	Average weight of total feces (g)
CTL 10	76.2 ± 2.21	10.30 ± 3.21	14.2 ± 2.32	0.42 ± 0.12	0.54 ± 0.28
Loperamide 2	203.6 ± 4.71^*∗∗∗*^	2.67 ± 2.31^*∗∗∗*^	3.12 ± 2.34^*∗∗∗*^	0.08 ± 0.23^*∗∗∗*^	0.12 ± 0.87^*∗∗∗*^
BLE 200	86.9 ± 2.42^*∗*^	4.33 ± 2.12^*∗∗∗*^	5.36 ± 1.32^*∗∗∗*^	0.22 ± 0.32^*∗∗∗*^	0.28 ± 0.65^*∗∗∗*^
BLE 400	107.3 ± 1.85^*∗∗∗*^	3.67 ± 1.39^*∗∗∗*^	6.23 ± 2.14^*∗∗∗*^	0.18 ± 0.85^*∗∗∗*^	0.33 ± 0.85^*∗∗∗*^
BLP 200	89.38 ± 3.28^*∗*^	4.67 ± 2.54^*∗∗∗*^	5.38 ± 3.21^*∗∗∗*^	0.28 ± 0.12^*∗∗∗*^	0.31 ± 0.60^*∗∗∗*^
BLP 400	110.57 ± 1.32^*∗∗∗*^	4.00 ± 2.36^*∗∗∗*^	6.56 ± 1.65^*∗∗∗*^	0.22 ± 0.45^*∗∗∗*^	0.28 ± 0.48^*∗∗∗*^
BLC 200	96.23 ± 3.21^*∗∗*^	6.00 ± 3.12^*∗∗*^	8.39 ± 2.31^*∗∗*^	0.26 ± 0.18^*∗∗∗*^	0.39 ± 0.28^*∗∗∗*^
BLC 400	84.85 ± 2.31^*∗*^	5.67 ± 2.14^*∗∗∗*^	8.32 ± 3.24^*∗∗*^	0.24 ± 0.38^*∗∗∗*^	0.32 ± 0.83^*∗∗∗*^
BLA 200	90.43 ± 1.38^*∗∗*^	7.33 ± 2.14^*∗*^	11.23 ± 1.78^*∗∗*^	0.37 ± 0.88^*∗*^	0.52 ± 0.62
BLA 400	89.32 ± 2.31^*∗∗*^	7.00 ± 2.68^*∗*^	9.27 ± 1.88^*∗∗*^	0.32 ± 0.56^*∗*^	0.48 ± 0.38

Values are expressed as mean ± SEM (*n* = 5). CTL: negative control (1% tween 80 in water), STD: positive control (loperamide at 5 mg/kg body weight). ^*∗∗∗*^*p* < 0.001, ^*∗∗*^*p* < 0.01, ^*∗*^*p* < 0.05 compared to control compared to negative control).

**Table 5:** The binding affinity of the screened phytochemicals and standard drugs to the selected proteins.

Criteria	Compounds	PubChem CID	Antidiarrheal	Antioxidant	Antibacterial	Cytotoxic
			5ZHP	1R4U	1XFF	10G5
Isolated phytochemicals	Linolenic acid	5280934	−5.3	−5.4	−4.6	−6.0
	Oleic acid	445639	−4.9	−4.5	−4.6	−6.2
	Phytol	5280435	−5.6	−4.7	−5.0	−5.9

Standard drugs	Vincristine	5978	—	—	—	−9.1
	Loperamide	3955	−7.7	—	—	—
	Amoxicillin	33613	—		−6.5	—
	Ascorbic acid	54670067	—	−5.2	—	—

5ZHP: M3 muscarinic acetylcholine receptor, 1R4U: urate oxidase, 1XFF: glutaminase domain of glucosamine 6-phosphate synthase, and 10G5: crystal structure of human cytochrome P450 2C9.

**Table 6:** The absorption, distribution, metabolism, excretion, and toxicological properties of the isolated compounds.

Compounds	M.W (g/mol)	HBA	HBD	LogP (o/w)	M.R	BBB permeability	Violation score
Linolenic acid	278.43	2	1	5.66	88.99	No	1
Oleic acid	282.46	2	1	4.01	89.94	No	0
Phytol	296.53	1	1	4.77	98.94	No	1

M.W: molecular weight; HBA: hydrogen bond acceptor; HBD: hydrogen bond donor; LogP: lipophilicity; M.R: molar refractivity; BBB: blood-brain barrier permeability.

## Data Availability

All analyzed data during this research are included in the published manuscript. The generated datasets during this research are not publicly available though they can be providable from the corresponding author upon reasonable request.

## Supplementary Materials

Supplementary Materials The supplementary file contains the protocol for the isolation of the bioactive secondary metabolites from *Blumea lacera* (Burm.f.) DC. (Family: Asteraceae). In the protocol, a detailed description of a collection of the plant, preparation, and extraction of the plant material, chromatographic techniques, and isolation of phytochemicals have been portrayed. Proton NMR spectra of each reported compound (Linolenic acid, Oleic acid, and Phytol) have also been included in the file.
