# Supplementary material for: Chemico-Biological Profiling of Blumea lacera (Burm.f.) DC. (Family: Asteraceae) Provides New Insights as a Potential Source of Antioxidant, Cytotoxic, Antimicrobial, and Antidiarrheal Agents
Source: Evid Based Complement Alternat Med. 2022 Aug 12;2022:2293415. doi: 10.1155/2022/2293415 (PMC9391110; doi:10.1155/2022/2293415)
Supplement: Supplementary Materials — The supplementary file contains the protocol for the isolation of the bioactive secondary metabolites from Blumea lacera (Burm.f.) DC. (Family: Asteraceae). In the protocol, a detailed description of a collection of the plant, preparation, and extraction of the plant material, chromatographic techniques, and isolation of phytochemicals have been portrayed. Proton NMR spectra of each reported compound (Linolenic acid, Oleic acid, and Phytol) have also been included in the file. [file 2293415.f1.docx]

The supplementary file contains the protocol for the isolation of the bioactive secondary metabolites from *Blumea lacera*. In the protocol, a detailed description of a collection of the plant, preparation, and extraction of the plant material, chromatographic techniques, and isolation of phytochemicals have been portrayed. Proton NMR spectra of each reported compound (Linolenic acid, Oleic acid, and Phytol) have also been included in the file.

**Scheme for Isolation of reported phytochemicals from the methanol extract of *Blumea lacera* (Burm.f.) DC.**

**Collection and Preparation of the Plant Material**

The whole plant of *Blumea lacera* was collected from Gazipur, Bangladesh in May 2019. The plant was identified by an expert from Bangladesh National Herbarium (BNH) and a voucher specimen was deposited for collection. The accession number of *Blumea lacera* is 55315.

The plant parts were cleaned properly. The parts were cut into small pieces and subjected to shade drying for 1 week. The dried material was then crushed into coarse powder by a high-capacity grinding machine with proper care.

**Extraction of the Plant Material**

About 750 g of the powdered material of *Blumea lacera* was taken in a clean, amber-colored bottle and soaked in distilled methanol for 15 days with occasional shaking and stirring. After two weeks of cold extraction, the whole mixture was filtered by a cotton plug in the large funnel and the volume of the filtrate was then reduced using a Buchii Rotavapor.

This process was done multiple times over 6 days and dried extracts were collected in the same beaker. The weight of the cumulative extract of *Blumea lacera* was 25g.

**Vacuum Liquid Chromatography of the Crude Extract**

Vacuum liquid chromatography is used for the initial rapid fractionation of the crude extract. The method and apparatus described by Pelletier *et al.*, 1986 were followed here (Pelletier et al., 1986). The column was packed under vacuum with fine VLC grade silica (Kiesel gel 60H) up to a height of 6 cm. The column was washed with pet-ether to ensure compact packing. The sample was prepared by dissolving it into methanol and mixing it with silica with subsequent drying. The dried sample was applied to the top of the column and the elution was commenced with pet ether polarity of which was gradually increased by adding more polar solvents like chloroform, ethyl acetate, and methanol.

**Table 1: Different solvent systems used for VLC of crude Methanol extract of *Blumea lacera***

| Beaker no. | Solvent system | Volume collected |
| --- | --- | --- |
| 1,2 | 100% Hexane | 100 ml |
| 3 | 2% EA in Hexane | 100 ml |
| 4 | 5% EA in Hexane | 100 ml |
| 5 | 8% EA in Hexane | 100 ml |
| 6 | 10% EA in Hexane | 100 ml |
| 7 | 12% EA in Hexane | 100 ml |
| 8 | 15% EA in Hexane | 100 ml |
| 9 | 20% EA in Hexane | 100 ml |
| 10 | 25% EA in Hexane | 100 ml |
| 11 | 28% EA in Hexane | 100 ml |
| 12 | 30% EA in Hexane | 100 ml |
| 13 | 32% EA in Hexane | 100 ml |
| 14 | 35% EA in Hexane | 100 ml |
| 15 | 38% EA in Hexane | 100 ml |
| 16 | 40% EA in Hexane | 100 ml |
| 17 | 42% EA in Hexane | 100 ml |
| 18 | 45% EA in Hexane | 100 ml |
| 19 | 48% EA in Hexane | 100 ml |
| 20 | 55% EA in Hexane | 100 ml |
| 21 | 60% EA in Hexane | 100 ml |
| 22 | 65% EA in Hexane | 100 ml |
| 23 | 70% EA in Hexane | 100 ml |
| 24 | 80% EA in Hexane | 100 ml |
| 25 | 90% EA in Hexane | 100 ml |
| 26 | 100% EA | 100 ml |
| 27 | 1% MeOH in EA | 100 ml |
| 28 | 2% MeOH in EA | 100 ml |
| 29 | 5% MeOH in EA | 100 ml |
| 30 | 10% MeOH in EA | 100 ml |
| 31 | 20% MeOH in EA | 100 ml |
| 32 | 30% MeOH in EA | 100 ml |
| 33 | 50% MeOH in EA | 100 ml |
| 34 | 70% MeOH in EA | 100 ml |
| 35 | 90% MeOH in EA | 100 ml |
| 36 | 100% MeOH | 100 ml |

EA= Ethyl Acetate, MeOH= Methanol

**Gel Permeation Chromatography (GPC)**

The column was packed with Sephadex (LH-20). At first, Sephadex was soaked in Chloroform for 24 hours for proper swelling. The slurry was then added to a glass column having 55 cm in length and 2.5 cm in diameter. The sample was dissolved in 20% pet ether in chloroform and subsequently applied on the top of the column with the help of a Pasteur pipette. The column was then eluted with the same solvent mixture and then followed by 10% pet ether in chloroform and 100% Chloroform. The elutes obtained were collected in numbered test tubes. Depending upon the TLC screening, similar fractions were mixed, and selected column fractions were subjected to further investigation (Gellerstedt, 1992). The solvent systems used as mobile phases in the analysis of the extract are listed in Table 2.

**Table 2: The solvent systems used as mobile phase in GPC**

| Serial no. | Solvent system |
| --- | --- |
| 1 | 20% PC (Petroleum Ether: Chloroform =20:80) |
| 2 | 10% PC (Petroleum Ether: Chloroform =10:90) |
| 3 | 100% Chloroform |
| 4 | 1% MC (Methanol: Chloroform =1:99) to  99% MC (Methanol: Chloroform =99:1) |
| 5 | 100% Methanol |

**Thin Layer Chromatography (TLC)**

TLC aluminum plates (20X20 cm) pre-coated with silica gel (Keiselgel 60 PF 254) was used for qualitative purpose using various solvent systems. In this method, a small spot of sample solution was applied to the silica gel layer just above 2cm from the edge of the plate using a capillary tube. In the meantime, the desired solvent was poured into the TLC tank and allowed for some time to saturate the compartment. Then, the plate was placed carefully into the tank without the applied sample being dipped into the solvent. Then the sample was separated as the solvent run upward from the bottom of the plate (Wagner and Bladt, 1996).

**Preparative Thin Layer Chromatography (PTLC)**

PTLC is a routinely employed method for the final purification of compounds. Pre-coated TLC aluminum plates coated with silica gel (Keiselgel 60 PF 254) were used for preparative TLC. The sample was dissolved in a small amount of solvent and applied to the plate as a thin band at 2 cm from the edge of the plate. The plates were developed in an appropriate solvent system previously determined by TLC. Then the plates are allowed to dry, and the compounds were visualized using UV-light or reagent spray. Then the compounds were scrapped and eluted with an appropriate solvent (Sherma and Fried, 1987).

**Visualization / Detection of the Compounds**

Different methods were applied for the detection of the compounds resolved on the developed chromatogram. They are described below:

**Visual detection:** The developed chromatogram was examined visually to detect the presence of colored compounds.

**UV light:** The developed chromatogram was observed under UV light (366 nm and 254 nm) and glowing and quenched spots were marked.

**Spray reagents**: Two different spray reagents were used primarily for detection purposes. The reagents are:

**Vanillin-Sulfuric acid reagent:** The developed chromatogram was sprayed with 1% Vanillin-Sulfuric acid reagent and then heated at 110ᵒC for 10 minutes and colors imparted by different compounds were observed.

**Dragendorff’s reagent:** It is a solution of Bismuth Subnitrate and Potassium Iodide in Acetic Acid and water. Alkaloids and other Nitrogen-containing compounds produce orange to red spots with this reagent.

**Isolation of Compound 1**

Sephadex fractions 18-29 of VLC fractions 11-12 yielded compound **1.** It was collected as a colorless mass and was isolated by PTLC using solvent ratio Hexane: Chloroform = 80:20. The Rf value was found to be 0.62 and it showed purple color upon spraying Vanillin Sulfate.

**Isolation of Compounds 2 & 3**

Sephadex fractions 1-3 of VLC fractions 13-14 yielded compound **2 & 3.** It was collected as a light yellowish liquid and was isolated by PTLC using solvent ratio Hexane: Chloroform = 90:10. The Rf value was found to be 0.51 and it showed pale yellow color upon spraying Vanillin Sulfate, which turned black after heating.

**Reference:**

1. Pelletier, S. W., Chokshi, H. P., & Desai, H. K. (1986). Separation of diterpenoid alkaloid mixtures using vacuum liquid chromatography. Journal of natural products, 49(5), 892-900.
2. Gellerstedt, G., 1992. Gel permeation chromatography. In Methods in lignin chemistry (pp. 487-497). Springer, Berlin, Heidelberg.
3. Wagner, H. and Bladt, S., 1996. Plant drug analysis: a thin layer chromatography atlas. Springer Science & Business Media.
4. Sherma, J. and Fried, B., 1987. Preparative thin layer chromatography. In Journal of Chromatography Library (Vol. 38, pp. 105-127). Elsevier.


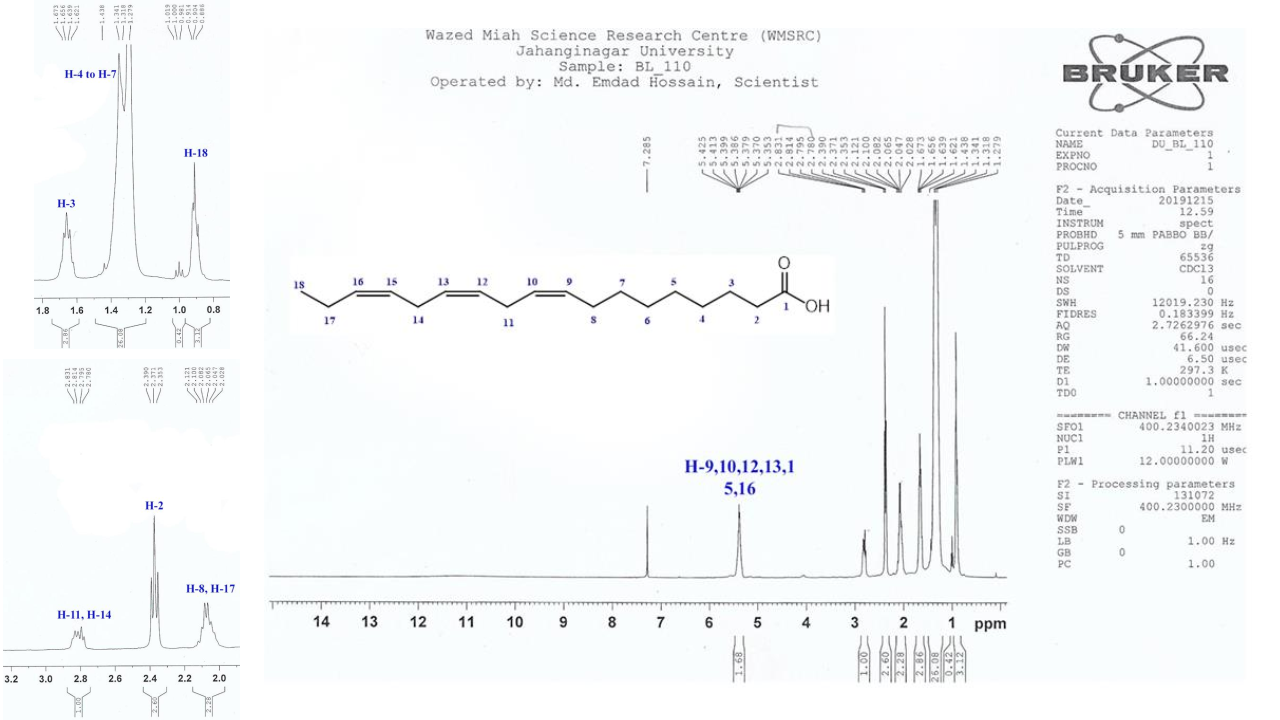


**Figure 1:** Proton NMR spectrum of isolated phytochemical Linolenic Acid from *B. lacera*


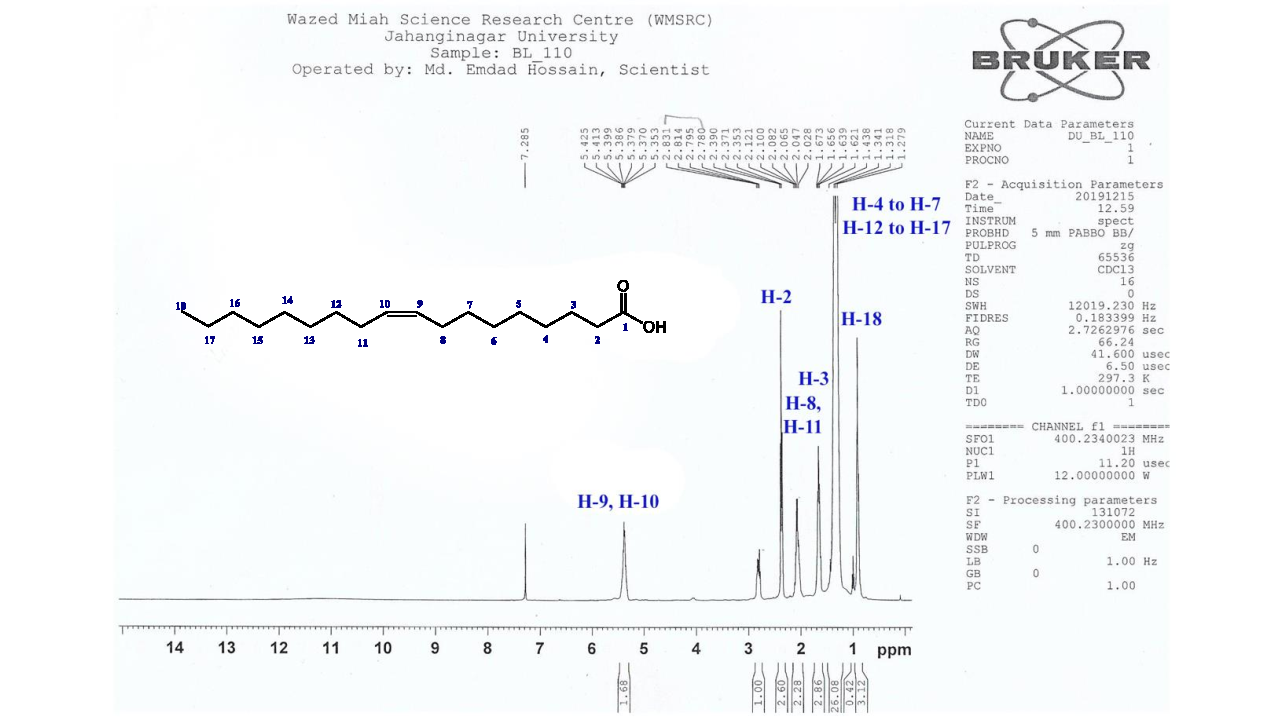


**Figure 2:** Proton NMR spectrum of isolated phytochemical Oleic Acid from *B. lacera*


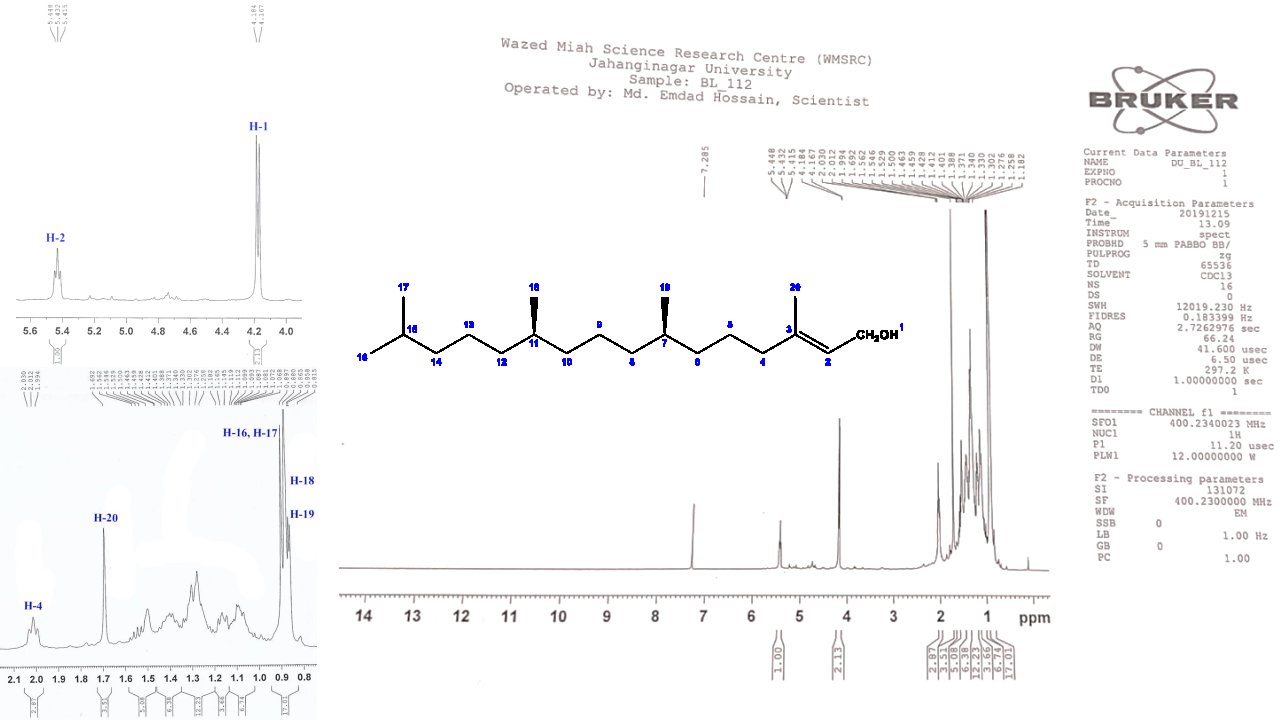


**Figure 3:** Proton NMR spectrum of isolated phytochemical Phytol from *B. lacera*
